# Monitoring within non-native ungulate exclosures documents the inherent size of *Crocanthemum greenei* (Cistaceae)

**DOI:** 10.3897/phytokeys.70.9363

**Published:** 2016-09-20

**Authors:** Tyler M. Dvorak, Amy E. Catalano, C. Matt Guilliams

**Affiliations:** 1 Catalina Island Conservancy, P.O. Box 2739, Avalon, CA 90704; 2 Santa Barbara Botanic Garden, 1212 Mission Canyon Road, Santa Barbara, CA 93105

**Keywords:** Cistaceae, Crocanthemum greenei, Helianthemum, Santa Catalina Island, stem measurement

## Abstract

*Crocanthemum greenei* (B.L.Rob.) Sorrie (Cistaceae), a perennial sub-shrub, was measured as part of a demographic monitoring effort on Santa Catalina Island, California, USA (hereafter, Catalina). Introduced ungulate browsers remain present on Catalina. Consequently, many palatable plant taxa on the island are subject to and putatively limited by top-down browsing forces. Historically, introduced ungulates have also been present on each island throughout the range of *Crocanthemum greenei*. Habitat conservation work, resulting in the construction of ungulate exclosures on Catalina, has now allowed us to measure individuals in their mature, non-browsed form. The published value for *Crocanthemum greenei* stem (height) is usually 15–30 cm. While the original description hints at a greater potential size, recent descriptions appear to be influenced by observations made during the decades when plants would have been impacted by introduced ungulate herbivores. Here we present stem measurements of 81 adult individuals, with a median of 49 cm and an interquartile range of 42–56 cm. These measurements suggest an expanded stem (height) range of 15–60 cm better describes the taxon and shed light on the historical impacts of invasive ungulates across the islands and those continuing on Catalina.

## Introduction

*Crocanthemum greenei* (B.L.Rob.) Sorrie (Cistaceae) is a perennial sub-shrub endemic to the Channel Islands of southern California. It is currently known from Santa Catalina (hereafter, Catalina), Santa Cruz, and Santa Rosa islands. It was also documented on San Miguel Island, but is now considered extirpated from that locale (Thorne 1967, McEachern 2010). *Crocanthemum greenei* is federally listed under the Endangered Species Act as threatened. It has presumably declined due to the browsing impacts of introduced ungulates throughout its range. Eradication efforts have removed all populations of introduced ungulates across the entire Channel Islands archipelago, with the exception of mule deer (*Odocoileus hemionus*) and American bison (*Bison bison*) on Catalina.

**Figure 1.**
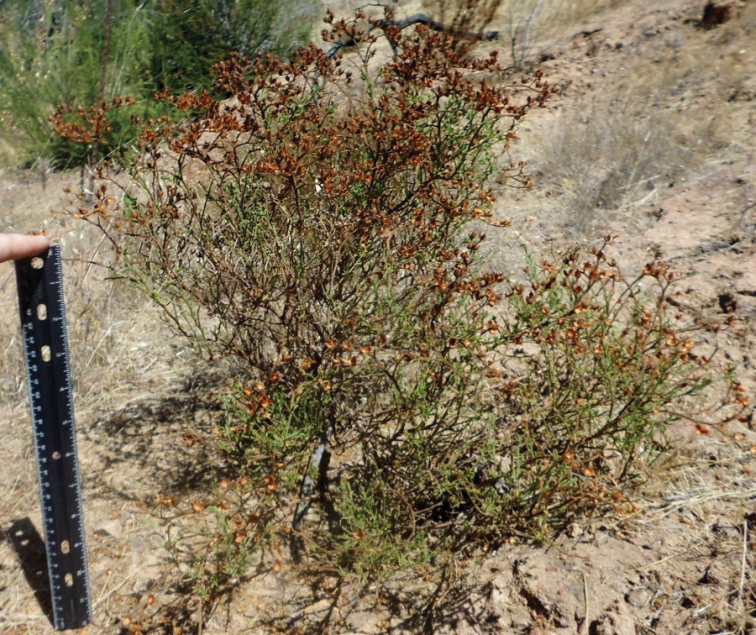
An individual from the current monitoring effort for *Crocanthemum greenei*. This particular individual, growing within an exclosure, had an initial stem measurement of 44 cm and is representative of size for non-browsed individuals.

A number of ungulate exclosures were constructed from 2008–2011 and have been maintained on Catalina for habitat recovery following wildfires and for focused conservation of select plant taxa. *Crocanthemum greenei* is present within six of these exclosures. We monitored 81 mature individuals within the exclosures as part of an overall island-wide demographic monitoring and study effort that is underway for the species. We measured the main stem of each plant as part of our monitoring protocol. All individuals were measured with a standardized method by the same researcher (A.E.C). A measuring tape was stretched from the base of the main stem to its tallest point, excluding the inflorescence. Photographs were taken of every individual with a standard ruler held or leaning next to the plant for scale (Fig. 1). This documented the physiognomy of each individual measured and permitted later inspection, when necessary.

## Results

**Figure 2.**
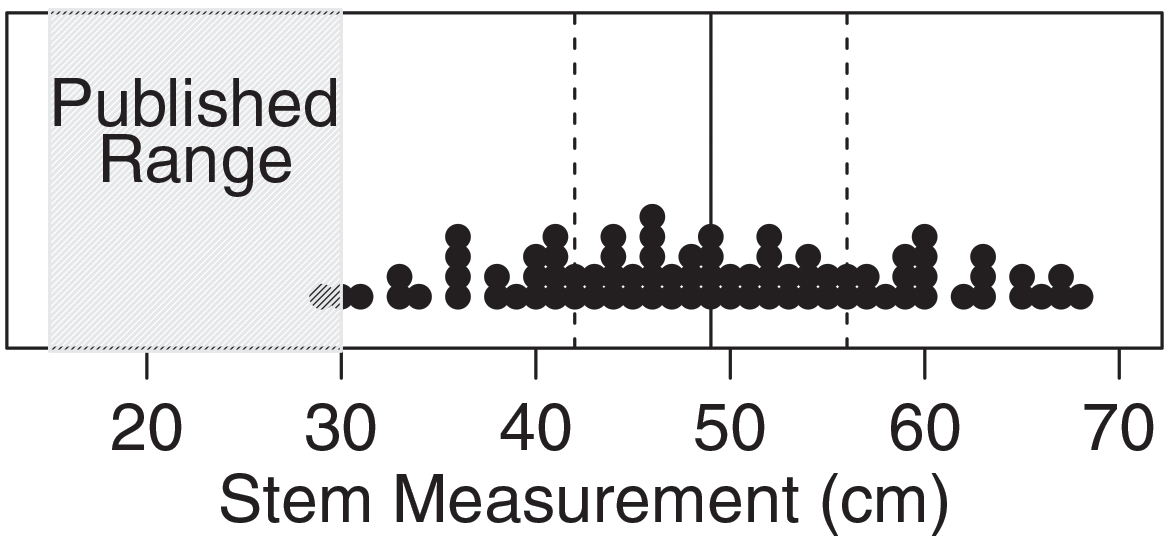
A plot of each exclosure individual in relation to the published stem range of 15–30 cm. The median of the dataset (49 cm) is marked with a solid line and the interquartile range (42–56 cm) lies between the dotted lines.

*Crocanthemum greenei* stem height measurements within exclosures (*n* = 81) had a range of 29 cm to 68 cm (Fig. 2). Median and mean were nearly identical at 49.00 cm and 49.02 cm, respectively. The interquartile range of the measurements was 42–56 cm. The most recent taxonomic treatment of *Crocanthemum greenei* states that stem length is 15–30 cm (Baldwin et al. 2012, Sorrie and Rosatti 2014). These data show that stem (height) of *Crocanthemum greenei* can be at least as tall/long as 60 cm in the absence of introduced herbivores.

## Discussion

**Figure 3.**
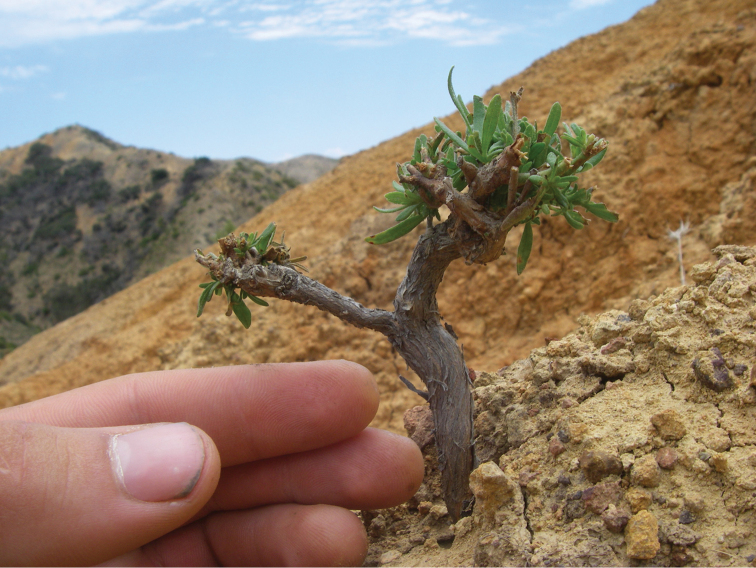
A severely browsed individual from a population not protected by exclosure fencing.

Our measurement data show that *Crocanthemum greenei* may grow substantially taller than previously reported in the absence of browsing by introduced ungulates. Furthermore, during our monitoring efforts we have consistently documented individuals outside of exclosures exhibiting severely browsed growth forms (Fig. 3), which lends evidence toward browsing as the limiting factor to achieving these sizes rather than the possibility of morphological variation due to external factors such as between-year climate (Dvorak and Catalano 2016). The measurements summarized here represent well documented, quantitative evidence of the natural growth form of *Crocanthemum greenei* when not modified by introduced browsers. We feel a particularly significant point is that introduced species have likely obscured our understanding of some basic aspects of the natural history of this rare, native island-endemic plant.

*Crocanthemum greenei* was first recognized as distinct from co-occurring *Crocanthemum scoparium* (Nutt.) Millsp. by Edward L. Greene in 1886. Greene named the new species from Santa Cruz Island *Helianthemum occidentale* Greene, but this name was already in use for a European plant (*Helianthemum occidentale* Nyman) and was therefore an illegitimate homonym. Regardless, Greene described the new species as suffrutescent, “a foot or more high”, and as having an inflorescence densely covered with glandular-viscid hairs; this latter feature distinguished it from *Helianthemum scoparium*, which is glabrous or with sparse short-glandular hairs. In his Flora of North America treatment of *Helianthemum*, Robinson (1895) recognized the new plant as *Helianthemum greenei* Robinson, providing roughly the same plant height of “6 inches to more than foot in height”. Munz (1959) recognized the species in A California Flora, in which he described the plant as having stems 1–2 dm (10–20 cm) high, rarely to 3 dm high (30 cm). In their monograph on *Helianthemum*, Daoud and Wilbur (1965) describe the species as being 14–30 cm tall. Sorrie (2011) later transferred all western North American *Helianthemum* taxa to the genus *Crocanthemum* based on unpublished molecular phylogenetic evidence that *Helianthemum* s.l. is polyphyletic (Arrington 2004). In the most recent treatment of *Crocanthemum* in California (Sorrie and Rosatti 2014), stem height is given as 15–30 cm. Therefore, the prevailing view since the species was first described was of a plant between approximately 15 and 30 cm tall (stem height 10–20 cm in Munz). We hypothesize that the difference between the prevailing view described above and our observations of the species on Catalina is due to the recent exclusion of introduced herbivores from our study plots.

Since the original description of the species was made on the basis of plants collected on Santa Cruz Island, the primary ungulate impacts relative to those plants would have come from sheep. The first record of sheep introduction on Santa Cruz Island was in the mid-1850s and the first effects on the vegetation due to grazing were reported in 1875 (Hobbs 1980). This timeline places Greene’s original collection (1886) during the sheep-grazing period and after the effects of introduced herbivores on the landscape had been noted.

Recent conservation and restoration efforts on the Channel Islands have eradicated ungulates from Santa Rosa and Santa Cruz islands, and resulted in actions on Catalina Island including the creation of exclosure habitats where our measurements of *Crocanthemum greenei* were made. With browsing pressure removed in some portions of the historical range of *Crocanthemum greenei*, individuals of the species can now grow to reach their full, inherent size. Beyond initiating a revision of the morphological description of *Crocanthemum greenei*, we hope that these observations are suggestive of both the capacity for recovery of a rare, island-endemic plant and the continuing need to remove the remaining ungulates from its range, which would bring to completion a critical conservation action for the Channel Islands archipelago.
